# Key lines of discovery in myopia research

**DOI:** 10.1111/opo.13508

**Published:** 2025-04-08

**Authors:** Frank Schaeffel, Jeremy A. Guggenheim, Richard A. Stone, Christine F. Wildsoet

**Affiliations:** ^1^ Institute of Molecular and Clinical Ophthalmology Basel (IOB) Basel Switzerland; ^2^ Institute for Ophthalmic Research University of Tuebingen Tuebingen Germany; ^3^ School of Optometry and Vision Sciences Cardiff University Cardiff UK; ^4^ Department of Ophthalmology Perelman School of Medicine, University of Pennsylvania Philadelphia Pennsylvania USA; ^5^ Herbert Wertheim School of Optometry and Vision Science University of California Berkeley Berkeley California USA

## VISUAL CONTROL OF EYE GROWTH BY A LOCAL, RETINAL MECHANISM

Myopia was considered a largely inherited condition in the 1970s when it was found by accident that monkeys became myopic when their eyes were deprived of spatial vision. The implication of this observation, that sufficient spatial visual information is a necessary prerequisite for normal refractive development, was confirmed by cross‐sectional observations in children with unilateral visual distortion, for example, by ptosis or corneal opacity, and by experiments in chickens, tree shrews, mice and guinea pigs, as well as additional experiments in rhesus monkeys (1978–2006). When retinal image contrast and/or spatial detail were restricted, so‐called form deprivation myopia developed rapidly. It was to become the most frequently used paradigm in animal myopia model studies for many years. Evidence that a direct retinal–scleral pathway underlies these altered growth responses, and that the visual regulation of eye growth more generally came from three different lines of research: (1) that deprivation confined to a local retinal area induced ‘local’ myopia, as demonstrated by local eye shape changes in chickens, guinea pigs and monkeys (1987–2015). Furthermore, deprivation myopia develops after either (2) the optic nerve was first sectioned to isolate the eye from the brain (chickens, guinea pigs; 1988–2020) or (3) retinal ganglion cells (and epithelial transport) were silenced by intravitreal tetrodotoxin injection (chickens, tree shrews 1990–1995).

## CLOSED‐LOOP VISUAL (OPTICAL DEFOCUS) CONTROL OF EYE GROWTH

In chicks, it was found that ‘myopic eye growth’ could also be induced by imposing appropriate (negative) optical defocus (1988). Specifically, when defocus was imposed by lenses, axial eye growth rates increased to restore retinal image focus with the lenses still in place. That positive and negative lenses slowed and accelerated eye growth, respectively, provided the first convincing evidence of the bidirectional nature of eye growth regulation. Additionally, emmetropisation is a closed‐loop feedback system, as now confirmed in chicken, guinea pig, tree shrew, marmosets, rhesus monkey and humans (1988 to today). Defocus imposed on local regions of the retina elicited local compensatory eye growth in chicks, guinea pigs and monkeys, providing further indirect evidence of local control (1997–2013). Indeed, optic nerve lesioning studies also pointed to local retinal decoding of the sign of defocus (chicks, guinea pig). Bidirectional choroidal thickness changes have been a consistent observation across all species, providing further confirmatory evidence for local control, albeit indirect. Myopic defocus effects dominate over competing hyperopic defocus in chicks, at least in eyes with intact optic nerves. In both chicks and marmosets, different gene sets are activated, according to the sign of imposed defocus. In relation to underlying local mechanisms, identified genetic and molecular markers encoding the sign of defocus include ZENK (an acronym for the avian orthologue of the mammalian genes zif‐268, egr‐1, ngfi‐a and krox‐24), bone morphogenetic proteins (BMPs) and retinoic acid in chicks and guinea pigs. Distinct roles for ON and OFF pathways in the visual control of eye growth are also implied by a variety of studies (1989 to today). While the specific cue or cues used by the retina to decode the sign of the experienced defocus remains unresolved, there is evidence that chromatic aberration may be involved (1991–2024), with several lines of research pointing to puzzling differences in processing between emmetropic and myopic adult humans (2020 to today). Bifocal lens designs, in which defocusing power is limited to the lens periphery, were found to affect the central (on‐axis) refraction in rhesus monkeys, marmosets, guinea pigs, chicks and humans (2006–2014). These observations triggered the development of novel myopia‐inhibiting lenses, including ones that limited myopic defocus to the lens periphery, with the central area reserved for correcting the existing refractive error of the wearer. To date, both multifocal contact lenses and spectacles have been shown to slow the progression of myopia in randomised controlled trials. Nonetheless, there remain many unresolved issues, such as the unexplained significant inter‐subject differences in response to these anti‐myopia treatments, which slightly reduce spatial contrast, also slows myopia progression. Furthermore, why efficacy reduces over time, as evidenced in large clinical data sets, with potential implications for another unresolved issue, namely whether there is an ideal time to stop treatment.

## IDENTIFIABLE MOLECULAR AND BIOCHEMICAL MECHANISMS AND PATHWAYS IN THE RETINA, CHOROID AND SCLERA, AND PHARMACOLOGICAL IMPLICATIONS

Underlying myopia mechanisms have been studied extensively in animal models and, to a limited extent, in children. The roles of dopamine (DA), nitric oxide (NO), adrenergic agents, nicotinic cholinergic drugs, gamma‐aminobutyric acid (GABA) and adenosine receptor (ADOR) antagonists (7‐MX, caffeine and others) have been studied, with still others receiving more limited attention. Changes in retinal transmitter/neuromodulator levels have been linked to experimentally altered eye growth, with DA receiving the most attention. Both DA content and release were found to be decreased in eyes undergoing accelerated growth in response to retinal image degradation, which was reversible with DA supplementation (chick, mouse, monkey; 1988 to today). Following over two centuries of study using topical atropine as an anti‐myopia drug in humans, many muscarinic antagonists were found to inhibit deprivation myopia (1991 to today; chick, mouse, guinea pig, monkey and human), as well as lens‐induced myopia (chick, guinea pig), although the site of action of such drugs remains unresolved. Cycloplegia is not a prerequisite for atropine's efficacy, as once proposed, as slowed myopia progression with lower topical doses of atropine has been observed in large‐scale studies in children, despite reduced cycloplegic and mydriatic effects (1999 to today).

## OCULAR CIRCADIAN RHYTHMS AND LINKS TO NORMAL AND ABNORMAL EYE GROWTH AND MYOPIA DEVELOPMENT

An interaction was found between myopia development and diurnal cycles (1992 to today). With normal vision, axial eye growth and choroidal thickness display diurnal cycles, which show changes in amplitude and phase in eyes developing myopia in response to either retinal image degradation or negative lenses (chicks, guinea pigs). Intrinsic circadian clocks in the retina and choroid, along with the diurnal regulation of overall gene expression in these tissues, seem affected in experimental myopia in the chick; but the role of local clocks when inducing refractive errors needs to be clarified. Likewise, intraocular pressure also undergoes diurnal variation, with the rhythm being altered in myopic eyes (chick). Data linking changes in diurnal rhythms with refractive errors in humans are not only less convincing (2011) but also more challenging to collect. Also unresolved is the role of intrinsically photosensitive retinal ganglion cells (ip‐RGCs) in the regulation of eye growth/choroidal circadian rhythms (vs. the role of the central circadian clock), that is, as activators or modulators of eye growth control mechanisms.

## THE ROLE OF THE CHOROID IN EYE GROWTH—ACTIVE REGULATOR AND/OR BIOMARKER OF GROWTH DIRECTION?

Choroidal thickness changes accompany and potentially are precursors to, or modulators of, axial eye growth changes (chicken, marmoset, rhesus monkey, humans, 1995 to today), with changes in blood flow contributing, at least transiently (chicks, rhesus monkeys, marmosets, humans). Changes in choroidal thickness may be a response to changing metabolic demands. Inhibiting eye growth appears energetically costly, as evidenced by associated choroidal thickening, increased choroidal blood flow in humans and downregulation of scleral hypoxia‐inducible factor 1‐alpha (HIF‐1α) during hyperopia development and upregulation during myopia development in mice and guinea pigs. Yet contributions via increases in blood flow are transient, at least in the chick, mouse and guinea pig (2018 to today). Among other possible explanations is the proposed link between choroidal tension and emmetropisation (1986), with studies raising a potential role of the choroid in buffering/protecting the sclera from the physical expansion effect of intraocular pressure.

## DEVELOPMENTAL CHANGES IN REFRACTIVE ERROR AND OCULAR COMPONENTS AND THE EMERGING MYOPIA EPIDEMIC

The myopia epidemic is largely driven by changes in lifestyle and/or environment (1993–2021). Clinical epidemiology studies revealed marked increases in myopia prevalence worldwide, with South and East Asian countries especially affected. Notably, it was found that high levels of myopia developed in all ethnic groups growing up in Singapore (2006), and that the genetic risk loci for refractive error are shared across ancestries (2018). While not to the same extent, myopia prevalence figures have increased recently in many other regions of the world as well.

## OUTDOOR EXPOSURE, ENVIRONMENTAL LIGHTING, SPECTRA AND IMPLICATIONS FOR MYOPIA (AND ITS CONTROL)

Delays in the onset of myopia and lower levels of the condition have been linked with greater outdoor exposure in children (2007 to today), although the mechanism of these protective effects remains to be fully resolved. In chicks, outdoor sunlight and/or bright lighting was shown to inhibit eye growth, with eyes remaining hyperopic, and the effects of myopia‐inducing treatments attenuated. The properties of ambient lighting (illuminance, colour, timing) seemingly interact with the mechanisms controlling eye growth in chicks, mice, guinea pigs, tree shrews and rhesus monkeys, with some experimental evidence implicating non‐visual ip‐RGCs and melanopsin‐stimulated, dopamine release (leading to myopia inhibition in mice). When lighting wavelengths are constrained, species differences emerge, with red light inhibiting ‘myopic’ eye growth in tree shrews, rhesus monkeys and humans, but not in chicks and guinea pigs (2015 to today). Emerging myopia control clinical therapies present a further paradox; with foveal ‘repeated low level red laser’ (RLRL) therapy and appropriately timed blue light directed at the optic nerve head both reported to slow myopia progression. Spatially low pass filtered images, confined to the blue components and intended to simulate the combined effects of myopic defocus and chromatic aberration, inhibit eye growth in tree shrews and increase choroidal thickness in humans, albeit only in emmetropic subjects in the latter case.

## EDUCATION—MYOPIA LINK AND APPARENT CAUSAL ASSOCIATIONS

Evidence that the level of education is linked to the probability of becoming myopic and to the amount of myopia that develops (1988–2015) was confirmed by Mendelian randomisation and regression discontinuity techniques (2016–2020). Results may partly account for the reported association of myopia with near work and intelligence quotient (IQ). The associations with education and near work are also consistent with reported rises in both myopia prevalence and progression among children in China, particularly associated with the extended lockdown during the COVID‐19 pandemic.

## DEFINING THE BIOMARKERS, GENES AND CLINICAL CHARACTERISTICS OF PATHOLOGICAL MYOPIA AND RISK FACTORS

The acceptance of pathological myopia as an age‐related eye disease and leading cause of visual impairment represented significant progress, and its characterisation continues (1995 to today). Pathological myopia may occur as a by‐product of early onset, rapidly progressing myopia, triggered by environmental factors. It also may reflect the evolution of high myopia arising from a single gene mutation.

## REFRACTIVE ERRORS ARE HIGHLY HERITABLE (OR ARE THEY)?

Many genes with small effect sizes have been associated with myopia. Different genetic networks mediating eye growth stimulation and growth inhibition have also been identified (2002–2020). In chickens, selective breeding for low or high levels of deprivation myopia generated highly distinct populations after only two generations (2011), and an inbred line of guinea pigs was also found to be resistant to developing form deprivation myopia. Large, collaborative teams of researchers have identified hundreds of single nucleotide polymorphism (SNPs) genetic markers for myopia in humans, with pathway analyses from human genetics confirming links to light sensing, pigment biology and circadian rhythms. Gene–environment interactions can at least partly explain why refractive errors, such as myopia, can be highly heritable, as suggested by twin studies (1985–2011), and yet have a major contribution from lifestyle risk factors.

## DECLARATION

This is a short summary of 10 key lines of discovery in myopia research, as considered by the authors, and prepared on request by the chair of the International Myopia Conference (IMC) 2024, Professor Weizhong Lan.

## AUTHOR CONTRIBUTIONS

All authors were involved in writing the manuscript.

## CONFLICT OF INTEREST STATEMENT

The authors declare that they have no competing interests.

## Data Availability

No data were generated as part of the study.

